# Research progress on electronic phase separation in low-dimensional perovskite manganite nanostructures

**DOI:** 10.1186/1556-276X-9-325

**Published:** 2014-06-28

**Authors:** Lizhi Liang, Lei Li, Heng Wu, Xinhua Zhu

**Affiliations:** 1 National Laboratory of Solid State Microstructures, School of Physics, Nanjing University, Nanjing 210093, China

**Keywords:** Perovskite manganites, Low-dimensional nanostructures, Electronic phase separation

## Abstract

Perovskite oxide manganites with a general formula of R_1-*x*_A*x*MnO_3_ (where R is a trivalent rare-earth element such as La, Pr, Sm, and A is a divalent alkaline-earth element such as Ca, Sr, and Ba) have received much attention due to their unusual electron-transport and magnetic properties, which are indispensable for applications in microelectronic, magnetic, and spintronic devices. Recent advances in the science and technology have resulted in the feature sizes of microelectronic devices based on perovskite manganite oxides down-scaling into nanoscale dimensions. At the nanoscale, low-dimensional perovskite manganite oxide nanostructures display novel physical properties that are different from their bulk and film counterparts. Recently, there is strong experimental evidence to indicate that the low-dimensional perovskite manganite oxide nanostructures are electronically inhomogeneous, consisting of different spatial regions with different electronic orders, a phenomenon that is named as electronic phase separation (EPS). As the geometry sizes of the low-dimensional manganite nanostructures are reduced to the characteristic EPS length scale (typically several tens of nanometers in manganites), the EPS is expected to be strongly modulated, leading to quite dramatic changes in functionality and more emergent phenomena. Therefore, reduced dimensionality opens a door to the new functionalities in perovskite manganite oxides and offers a way to gain new insight into the nature of EPS. During the past few years, much progress has been made in understanding the physical nature of the EPS in low-dimensional perovskite manganite nanostructures both from experimentalists and theorists, which have a profound impact on the oxide nanoelectronics. This nanoreview covers the research progresses of the EPS in low-dimensional perovskite manganite nanostructures such as nanoparticles, nanowires/nanotubes, and nanostructured films and/or patterns. The possible physical origins of the EPS are also discussed from the signatures of electronic inhomogeneities as well as some theoretical scenarios, to shed light on understanding this phenomenon. Finally, the perspectives to the future researches in this area are also outlined.

## Review

### Background

Strongly correlated-electron materials, such as the rare-earth perovskite oxide manganites having a general formula R_1-*x*_A*x*MnO_3_, where R is a trivalent rare-earth element (e.g., La, Pr, Sm) and A is a divalent alkaline-earth element such as Ca, Sr, and Ba, have been attracting much attention because of their unusual electron-transport and magnetic properties, e.g., colossal magnetoresistance (CMR) effect [1-3], a sharp metal-insulator transition (MIT) as a function of temperature, electric field, magnetic field, light, hydrostatic pressure, strain, etc. [4-6]. Such MIT is also accompanied by a paramagnetic to ferromagnetic transition as the temperature is lowered. The competition between several interactions in the rare-earth perovskite oxide manganites makes that only small energy differences exist between the different possible phases of the system. As a result, the phase of the material can be tuned by various external perturbations, such as magnetic and electric fields, strain, and disorder. These perturbations may lead to the CMR effect and can be used for electronic phase control in manganite devices. Recently, there is strong experimental evidence to indicate that the rare-earth perovskite oxide manganites are electronically inhomogeneous, which consist of different spatial regions with different electronic orders [7-10], a phenomenon that is named as electronic phase separation (EPS). As an inherent electronic inhomogeneity, EPS has been widely reported in the rare-earth perovskite oxide manganites, and its size varies from nano to mesoscopic scales [11-15]. It has been recognized to be crucial for the CMR effect and the MIT in manganites, leading to the new applications of spintronics [9]. However, the presence of EPS raises many intriguing questions, e.g., what is the microscopic nature of the EPS? Why does it have such a large range of length scales from nanometers to micrometers? More importantly, is it responsible for the related physical properties such as CMR and high-Tc superconducting exhibited by the manganites and related oxide materials? Therefore, EPS is getting recognized as a phenomenon of importance in understanding the magnetic and electron transport properties of perovskite oxide manganites [16,17].

Recent advances in science and technology of perovskite oxide manganites have resulted in the feature sizes of the microelectronic devices based on perovskite oxide manganites entering into nanoscale dimensions. At nanoscale, perovskite oxide manganites exhibit a pronounced size effect manifesting itself in a significant deviation of the properties of low-dimensional structures from their bulk and film counterparts. For example, in the charge-ordered half-doped manganite nanoparticles, a robust charge ordering (CO) state and antiferromagnetic (AFM) ground state are significantly suppressed whereas a ferromagnetic (FM) ordering is exhibited [18-22]; meanwhile, there exists size-dependent exchange bias effect and glassy behaviors [23-26]. In the nanostructured patterns of (La,Pr,Ca)MnO_3_ (LPCMO) narrow strips (spatial confined system), several new transport features such as giant resistance jumps [27-30], reentrant M-I transitions [31], negative differential resistances, and intrinsic tunneling magnetoresistance [32,33] emerge, which are absent in the thin films and bulks. Furthermore, as the geometry size of the low-dimensional manganite nanostructures is further reduced to the characteristic EPS length scale (typically several tens of nanometers in manganites), the EPS is expected to be strongly modulated, leading to quite dramatic changes in functionality and more emergent phenomena [34]. Therefore, reduced dimensionality will open a door to the new functionalities in perovskite manganites and offer a way to gain new insight into the nature of EPS in the perovskite manganite system [35]. In the recent years, much progress has been made in understanding the physical nature of the EPS in low-dimensional perovskite manganite nanostructures both from experimentalists and theorists, which have a profound impact on the manganite oxide nanoelectronics. In this work, we review the major progress of the EPS in low-dimensional perovskite manganite nanostructures, which are based on the recent literatures about the EPS in perovskite manganite nanoparticles, nanowires/nanotubes, and nanostructured films and/or patterns. The possible physical origins of the EPS are also discussed from the signatures of electronic inhomogeneities as well as some theoretical scenarios to shed light on understanding this phenomenon. We end this review by providing our perspectives to the future research directions in this area.

### Research history of EPS and its signatures

The first report on the EPS in perovskite manganites was back to 1950s, where Wollan and Koehler carried out their pioneering neutron scattering studies of La_1-*x*_Ca*x*MnO_3_ (LCMO) [36]. They observed both FM and AFM peaks in the magnetic structure of LCMO by neutron scattering, and concluded that there is the simultaneous presence of FM and AFM phases in this material. Since that time, manganites had just begun to attract the interest of physicists. In 1950, Jonker and van Santen first reported the electrical and magnetic properties of manganites, and they found a ferromagnetic conducting phase below room temperature in La_1-*x*_Ca*x*MnO_3_ (0.2 < *x* < 0.4) [37,38]. Shortly afterward, Zener, Kanamori, Goodenough, and several others established the basic theoretical framework of the EPS that scientists use today [39].

Manganites and the phase separation effects they display fell out of fashion until the 1990s. Although significant magnetoresistance effects in single-crystal La_0.69_Pb_0.31_MnO_3_ were reported in 1969, there was no technological incentive for further pursuit [40]. What reignited manganite research was the 1994 discovery by Jin and collaborators that a several-tesla magnetic field could induce a 1,000-fold change in the resistance of a heat-treated epitaxial thin film of La_0.67_Ca_0.33_MnO_3_[41]. A dubbed CMR, this effect arises because the applied magnetic field drives a phase transition from an insulating paramagnet to a spin-aligned metal. Thus, as Jonker and van Santen reduced the temperature to reach the conducting spin-aligned phase, Jin and his colleagues applied a magnetic field.

Recently, Woodward et al. performed a neutron diffraction study of Nd_0.5_Sr_0.5_MnO_3_ and found that this material first became FM at 250 K, partially transforming to an A-type AFM phase at approximately 220 K, followed by a transformation of a substantial fraction to a CE-type AFM phase at approximately 150 K [42]. Their experimental results indicate that three phases (FM metallic and CE-AFM charge-ordered phases along with an A-type AFM phase) coexist at low temperatures, and the size scale of the inhomogeneities is at least in the mesoscopic range (a few hundred nanometres or more). Sub-micrometersized phase separation involving FM and charge-ordered AFM domains with a typical size of about 0.2 μm was found in La_0.625-*y*_Pr_*y*_Ca_0.375_MnO_3_ by transmission electron microscopy (TEM) [5]. At the same time, by using scanning tunneling spectroscopy (STM), Fäth et al. also found the evidence of electronic inhomogeneities in La_0.7_Ca_0.3_MnO_3_ below the FM transition temperature with a mesoscopic scale of about 0.2 μm, where the FM metallic domains are interspersed in insulating regions [43].

Mesoscopic phase separation with the length scale between 30 and 200 nm, arising from the comparable energies of the ferromagnetic metallic and antiferromagnetic insulating states, is just one extreme in the perovskite manganites [5]. Normally, the EPS with phases of different charge densities is expected to give rise to nanometer scale clusters because large phase separated domains would break up into small pieces due to the Coulomb interactions. For example, Mori et al. reported a nanoscopic length scale of the electronic inhomogeneity in thin films of the hole-doped side of (La,Ca)MnO_3_ by high-resolution TEM [44]. Similarly, in Bi_0.25_Ca_0.75_MnO_3_, Renner et al. also found nanoscopic charge-ordered and metallic domains which were correlated with the structural distortions [45]. Generally, microscopically homogeneous clusters are usually in the diameter size of 1 to 2 nm dispersed in an insulating or charge-localized matrix. For example, recently, De Teresa et al. [46] reported on the experimental evidence for the existence of nanoscopic phase segregation in the manganite compounds of (La_1-*x*_A_*x*_)_2/3_Ca_1/3_MnO_3_ (A = Y or Tb), in which the spontaneous formation of localized magnetic clusters with size of ~1.2 nm above the ferromagnetic ordering temperature was revealed by a combination of volume thermal expansion, magnetic susceptibility, and small-angle neutron scattering measurements. These nanosized magnetic clusters grew in size but decreased in numbers under an external applied magnetic field. Such a phase separation scenario bridges the gap between the double-exchange model and the lattice distortion models.

The signatures of EPS can be revealed by different techniques depending on the length scale on which it occurs. For mesoscopic phase separation, diffraction techniques can be used to reveal its distinct features since the size scale of the inhomogeneities is large enough to produce well-defined reflections in neutron and X-ray diffraction patterns [9]. However, for the nanoscopic electronic inhomogeneity in manganites, both TEM, high-resolution TEM and scanning transmission electron microscopy (STEM), and STM can be used to reveal the coexistence of nanoscopic charge-ordered (insulating) domains and the FM metallic domains, giving the local structural information at atomic level [5]. It is often difficult to identify EPS based on the magnetization and transport measurements because of the sensitivity of phase separation to magnetic fields. Thus, magnetic fields transform the antiferromagnetic insulating state to the ferromagnetic metallic state. However, transport measurements, under favorable conditions, can provide valuable information on phase separation.

### EPS in low-dimensional perovskite manganite nanostructures

Over the last decade, nanomaterials have received much attention from the scientific and engineering viewpoints. They exhibit different properties from those of bulk materials due to their small size and large surface-to-volume ratios, and become promising candidates for nanometer scale electronic, optical, and mechanical devices. Recent advances in science and technology of perovskite manganites have resulted in the feature sizes of perovskite manganite-based oxide electronic devices entering into nanoscale dimensions. As the spatial dimension of the low-dimensional manganite nanostructures is reduced to the characteristic EPS length scale, quite dramatic changes in their transport properties such as ultrasharp jumps of magnetoresistance, reentrant MIT, negative differential resistances, and intrinsic tunneling magnetoresistance could appear, which are believed to be caused in large part by the EPS in perovskite manganite nanostructures [27-33]. They have significant impacts on fabricating oxide-based novel devices. To better understand the EPS phenomenon in low-dimensional perovskite manganite nanostructures, in the past several years, various synthetic methods such as sol–gel technique [47], hydrothermal synthesis [48,49], electro-spinning process [50,51], template method [52-54], and lithographic techniques [27,29-31,33,34] have been developed to fabricate low-dimensional manganite nanostructures, such as manganite nanoparticles, nanowires/nanotubes, and nanostructured films/patterns. In addition, the new technologies to directly identify the EPS phenomenon in low-dimensional perovskite manganite nanostructures have been developed, such as scanning electron nanodiffraction [13], atomic-resolution STM [45], MFM [4,55], and electron holography [11]. In this section, we will review the recent progress on the EPS in low-dimensional perovskite manganite nanostructures.

### EPS in manganite nanoparticles

**Figure 1:**
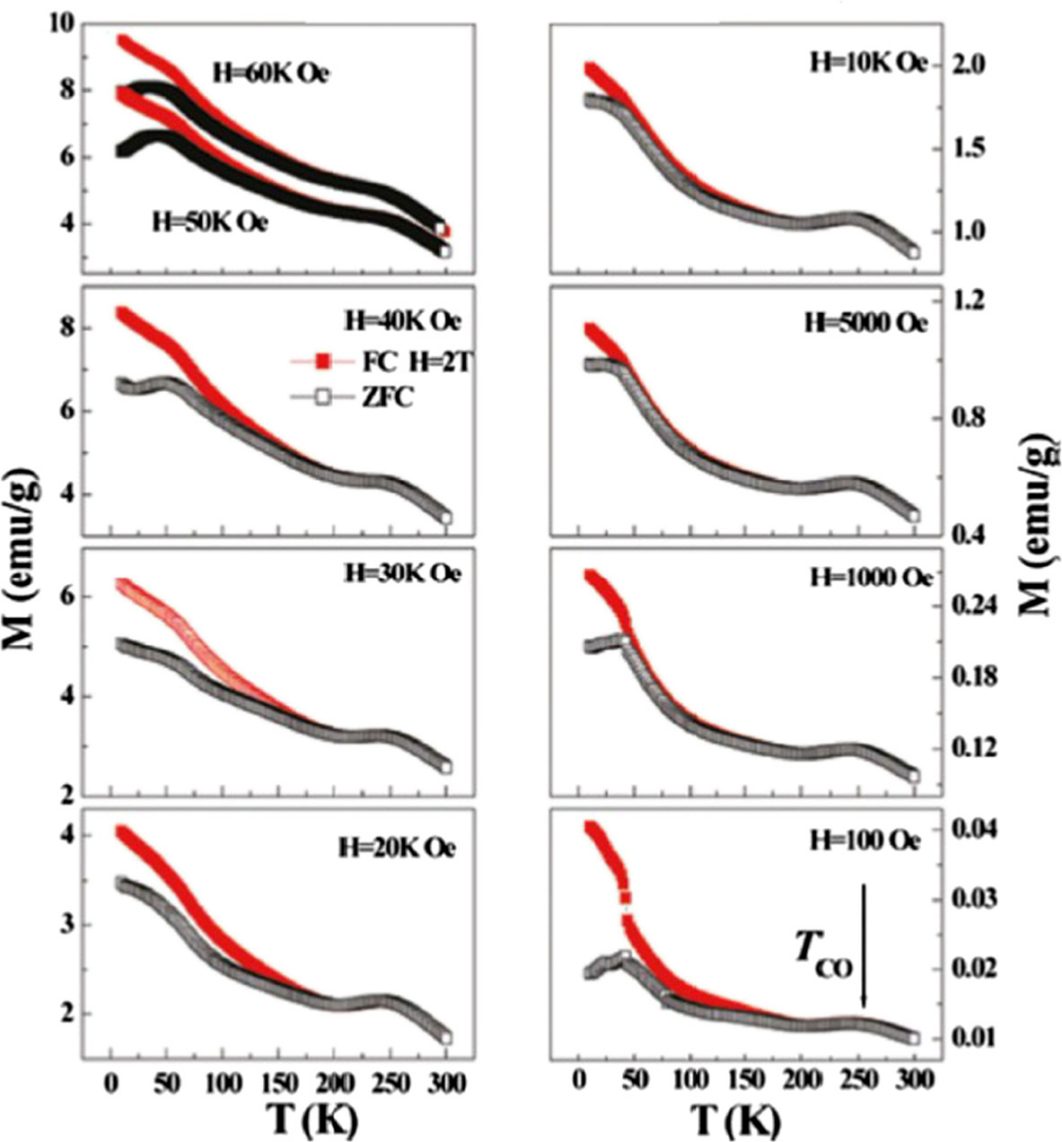
**Field cooled and zero field cooled magnetization of Pr**_**0.67**_**Ca**_**0.33**_**MnO**_**3 **_**nanoparticles.** Field cooled (closed symbols) and zero field cooled (open symbols) magnetization of Pr_0.67_Ca_0.33_MnO_3_ nanoparticles as a function of temperature, measured in the field ranges from 100 Oe to 60 kOe [57].

**Figure 2:**
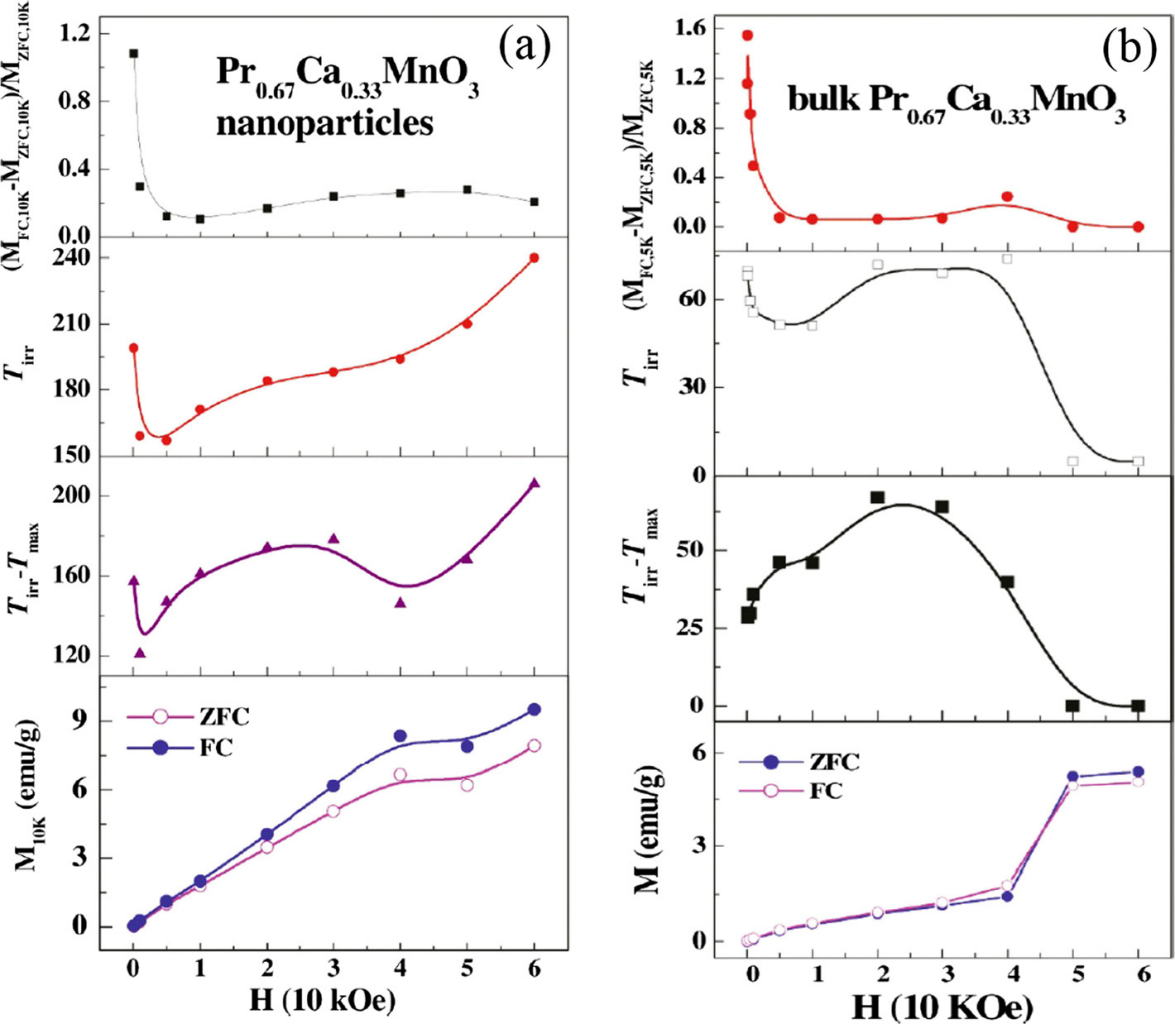
**Field dependence of the analyzed magnetization data for (a) Pr**_**0.67**_**Ca**_**0.33**_**MnO**_**3 **_**nanoparticles and (b) bulk counterpart [**[57]**].** The relative history dependence of the magnetization Δ*M* = (*M*_FC_-*M*_ZFC_)/*M*_ZFC_ was measured at 10 K for Pr_0.67_Ca_0.33_MnO_3_ nanoparticles and 5 K for bulk counterpart. *T*_irr_ is the irreversibility temperature; Δ*T* = *T*_irr_ - *T*_max_ is the difference between the irreversibility temperature and the temperature of the maximum ZFC magnetization. *M*_ZFC_ and *M*_FC_ at 10 K for Pr_0.67_Ca_0.33_MnO_3_ nanoparticles and 5 K for bulk counterpart.

EPS is an important phenomenon in CMR material, which leads to the new applications of spintronics. Along with the development of nanotechnology, the EPS phenomenon in CMR nanoparticles are received much attention. Recently, the evolution of the EPS with magnetic field in nanosized Nd_0.5_Ca_0.5_MnO_3_[19], La_0.25_Ca_0.75_MnO_3_[47], Pr_0.5_Ca_0.5_MnO_3_[21], La_0.2_Ca_0.8_MnO_3_[56], and Pr_0.67_Ca_0.33_MnO_3_[57] particles has been reported. For example, in nanosized Pr_0.67_Ca_0.33_MnO_3_ particles with average diameter of 100 nm, it was found that a sharp transition from AFM to FM did not occur even up to 60 kOe, as demonstrated in Figure 1[57]. The field dependence of the analyzed magnetization data for the Pr_0.67_Ca_0.33_MnO_3_ nanoparticles is shown in Figure 2[57]. As a comparison, the data for the bulk counterpart is also given out. It is clear that the evolution tend of Δ*M* is a little different from that of the bulk counterpart, i.e., first a sharp decrease and then an increase slowly up to 50 kOe. However, the irreversibility temperature (*T*_irr_) exhibits a very different change tend as compared with that of the bulk counterpart, which is sharply decreased from 100 to 5,000 Oe and then continually increased. The magnetization *M*_ZFC_ and *M*_FC_ are increased smoothly with increasing the magnetic field *H* up to 60 kOe but a step-like increase of *M*_ZFC_ and *M*_FC_ like in bulk counterpart is not observed. For *H* below 5,000 Oe, the first sharp decrease of the Δ*M T*_irr_, and weak decline of Δ*T* is attributed to the gradual conquest of the anisotropy of frozen spin and alignment with field, since the magnetic field is not large enough to induce the growth of the FM cluster. Due to the surface effect, the FM-like surface spins contribute additional moment, which leads to a large magnetization for nanoparticles as compared with bulk counterpart. However, due to the strong coupling between the surface spins and interface spins (which also deviate from AFM arrangement), the exchange field required to force a transition of surface spins and interface spins to full FM is approximately 5 × 10^6^ Oe [58]. As a consequence, even the field is increased up to 60 kOe, which can align the AFM core spins like for bulk, it is still not large enough to make the nanoparticles to be full FM configuration, thus leading to a slow increase of the Δ*M* and *T*_irr_[58]. The significant increase of the exchange bias field of the Pr_0.67_Ca_0.33_MnO_3_ nanoparticle as compared with the bulk counterpart can be attributed to the surface pressure and uncompensated surface spins.

Recently, the EPS in La_0.7_Sr_0.3_MnO_3_ nanoparticles synthesized by sol–gel process was also investigated by electron magnetic resonance (EMR) method [59]. The results showed that all the La_0.7_Sr_0.3_MnO_3_ nanoparticles (synthesized with different gelation agents) exhibited the following common features: (i) at the PM region, the EMR line was pure Lorentzian having a *g* value decreasing with increasing the temperature and *g* value reached 2 at around 350 K; (ii) when the temperatures are crossing Tc, the EMR lines changed their resonance fields (e.g., lineshapes and linewidths); (iii) all samples showed the coexistence of FM and PM signals within a wide temperature range below Tc; and the intensity of PM signal increased gradually as the temperature approached to Tc. The growth of PM phase was accompanied by a consequent decrease of FM signal intensity. Besides these common features, the EMR spectra of the measured samples also show several significant differences, which allow ones to investigate the origin of PS in these samples. It was found that the La_0.7_Sr_0.3_MnO_3_ nanoparticles synthesized with different gelation agents in sol–gel process exhibited different magnetic behaviors, and a sharp FM-PM transition was observed in the La_0.7_Sr_0.3_MnO_3_ nanoparticles synthesized with a combined agent of urea and trisodium citrate. These results also demonstrate that the synthesis conditions of perovskite manganite nanoparticles have an important role in their microstructure, magnetic properties, and phase separation behavior.

### EPS in manganite nanowires/nanotubes

**Figure 3:**
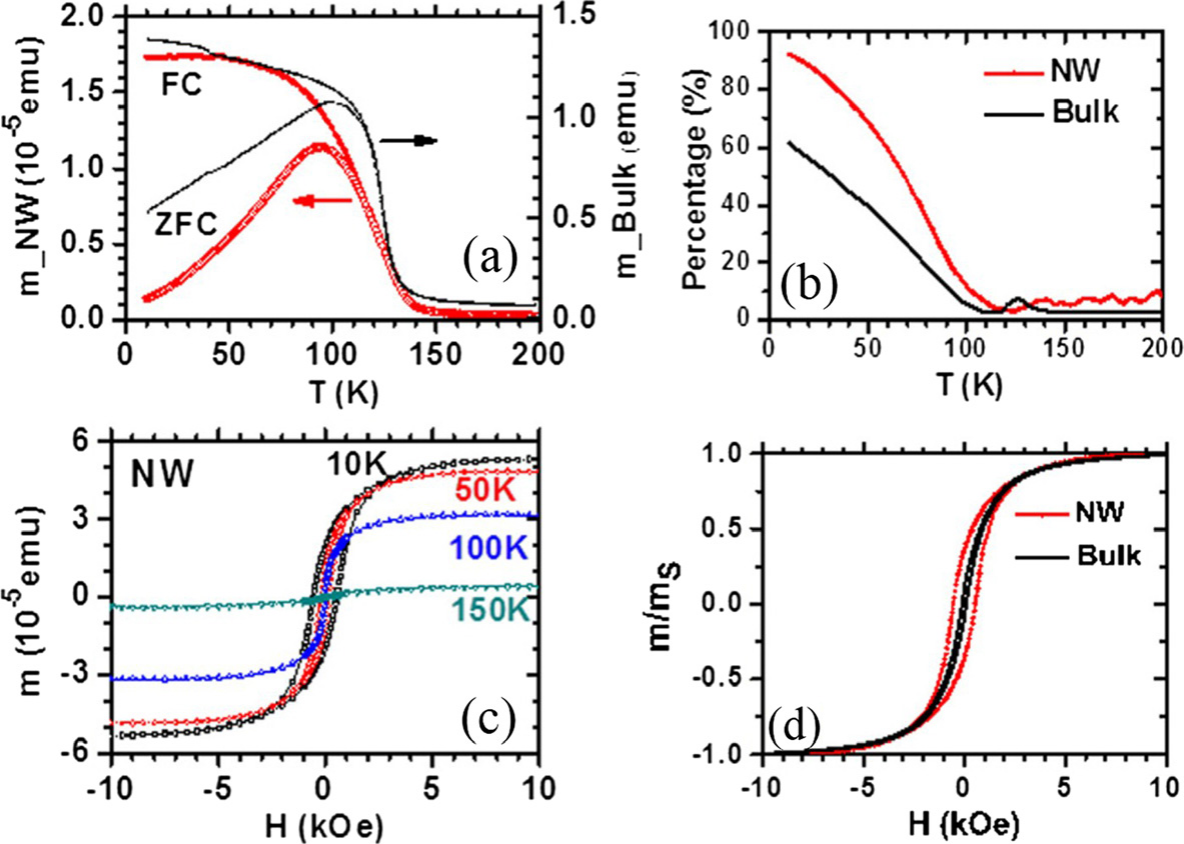
**Magnetic measurements of LPCMO/MgO nanowires. (a)** Magnetic moment versus temperature of the LPCMO/MgO nanowires (NW) and the LPCMO bulk polycrystalline sample after ZFC and FC [63]. The cooling field and the measuring field are both 200 Oe. **(b)** The percentage of the frozen phase defined as [*m*(FC)-*m*(ZFC)]/*m*(FC); **(c)** the field dependent magnetic moment of the LPCMO/MgO nanowires at different temperatures; and **(d)** the hysteresis loops of the nanowires and the bulk sample measured at *T* = 10 K.

One-dimensional manganite nanostructures that include nanowires, nanorods, and nanotubes have attracted rapidly growing interest due to their fascinating electrical and magneto-transport properties. They are emerging as important building blocks serving as interconnects and active components in nanoscale electronic, magnetic, and spintronic devices. It is expected that the manganite nanowires will exhibit an emerging magnetic and transport behaviors associated the EPS due to the strong electronic correlation under a spatial confinement in the case of nanowires [35]. Recently, theoretical calculations using the FM Kondo Hamiltonian have predicted that the intrinsic EPS persists in one-dimensional manganite nanostructures [60]. However, due to the growth difficulty in single-crystalline manganite nanowires, the EPS in manganite nanowires are not completely explored. Recently, it has been demonstrated that by utilizing MgO nanowires as the template one can grow the transition metal oxide core-shell nanowires with good single crystalline quality [61,62]. By the same method, Li et al. synthesized the single-crystalline La_0.33_Pr_0.34_Ca_0.33_MnO_3_ (LPCMO)/MgO core-shell nanowires with diameters about tens of nanometers [63]. Their structure and morphology characterizations confirm the epitaxial growth of La_0.33_Pr_0.34_Ca_0.33_MnO_3_ shell layers on MgO core layers. The magnetic measurements are shown in Figure 3[63]. As shown in Figure 3a, the ZFC curve and the FC curve of the LPCMO nanowires are split at a blocking temperature of *T*_b_ = 93 K when the temperature is decreased. Such a ZFC/FC deviation is very similar to that of the bulk polycrystalline LPCMO sample also shown in Figure 3a, and is due to the frozen of the magnetic moment. The differences between the ZFC and FC magnetic moments in the nanowire, defined as the frozen phase magnetic moment, is significantly larger than that in the bulk counterpart below the blocking temperature sample, as shown in Figure 3b. In bulk or thin film LPCMO, the frozen phase is generally regarded to be related to the phase competition between the FM metallic phase and the AFM-CO phase [64]. So, in the nanowires, the increased amount of frozen phase concentration could be reasonable due to the stronger phase competition in the low-dimensional system. Figure 3c,d displays the magnetic field dependence of the magnetic moments of the LPCMO nanowires and the bulk counterpart. As observed in Figure 3c both the saturation magnetic moment *m*_s_ and the coercivity *H*_c_ in the LPCMO nanowires were increased as the temperature was decreased, which was similar to that in bulk or thin-film manganites. However, the differences between the nanowire and the bulk sample were also observed. The *H*_c_ value of the LPCMO nanowires was much larger than that of the LPCMO bulk sample. For example, at *T* = 10 K, *H*_c_ is about 550 Oe in the nanowire but only about 100 Oe in the bulk sample as shown in Figure 3d. The larger *H*_c_ in the nanowires could be attributed to their stronger domain wall pinning at the boundaries of the separated AFM and FM phases caused by the EPS in the nanowires [65]. These observations suggest that the EPS with a stronger phase competition exists in the one-dimensional structure.

**Figure 4:**
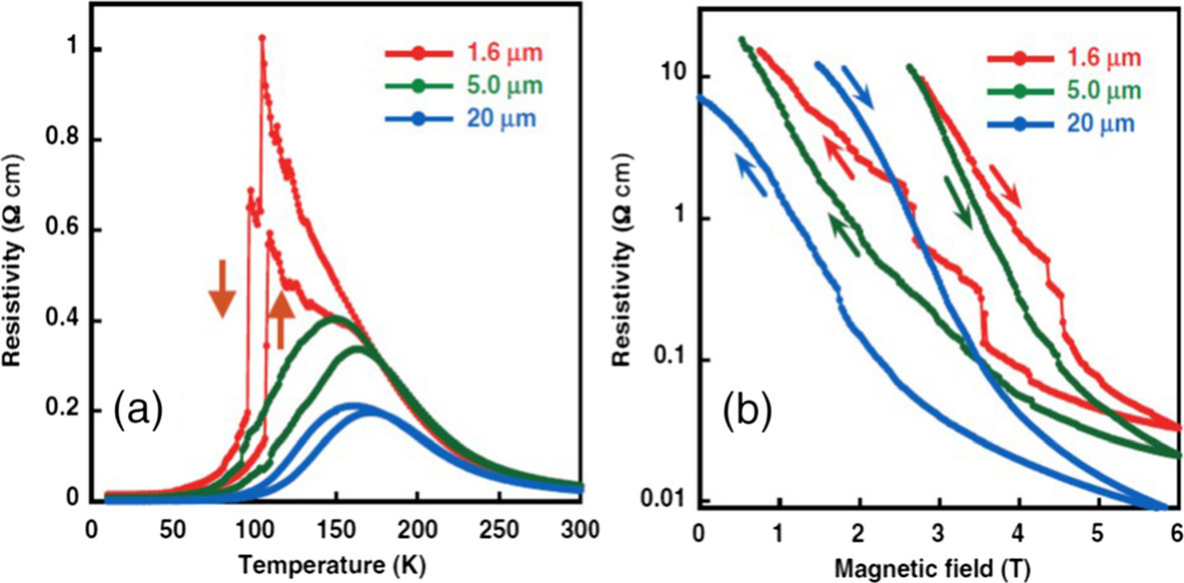
**Resistivity versus temperature curves and resistivity vs. magnetic field curves. (a)** Resistivity versus temperature (R-T) curves for the LPCMO wires under a 3.75-T magnetic field [27]. Arrows indicate the direction of the temperature ramp. The R-T curves all exhibit hysteresis behavior in cooling-warming cycles, which is consistent with the coexistence of ferromagnetic metal and charge-ordered insulator domains in the LPCMO system. The MIT is rather smooth for both the 20-μm and the 5-μm wires. Ultrasharp and giant steps are clearly visible for the 1.6-μm wire. **(b)** Resistivity vs. magnetic field curves for the LPCMO wires measured at 110 K. Sudden step-like jumps are again visible in the 1.6-μm wire. Arrows indicate the sweeping directions of the magnetic field for each curve.

**Figure 5:**
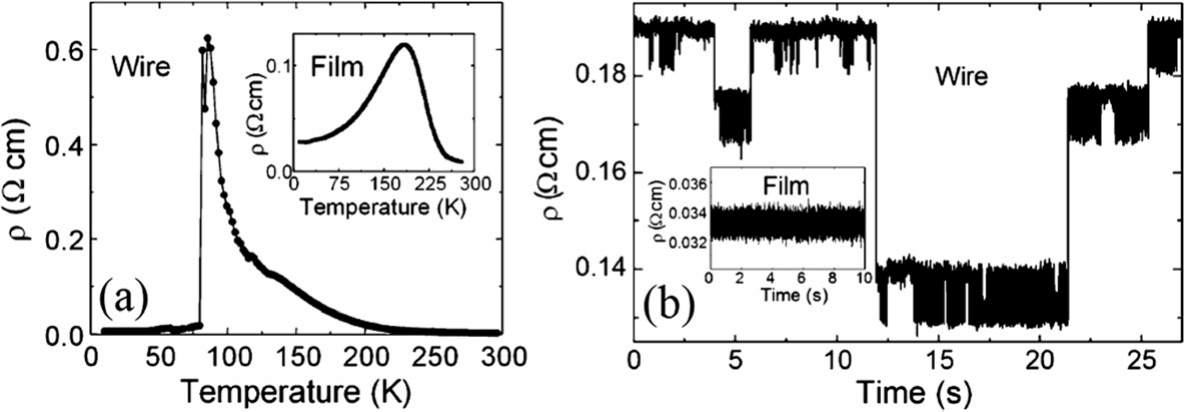
**Time-dependent resistivity measurements. (a)** Wire shows abrupt drop in resistivity at the MIT transition while the film shows a smooth transition (inset) [29]. **(b)** Resistivity of a wire when held at the transition temperature shows clear jumps associated with single electronic domain fluctuations. This behavior is not observed in the film, which only exhibits white noise (inset).

To better understanding the role of EPS in the emergent transport behaviors of manganite one-dimensional structures, some manganite wires are also fabricated from single thin film by optical lithography, where the width of the manganite wires is reduced to a scale on the order of the inherent phase separation. As one example, LPCMO is chosen as a representative model system due to its sub-micrometer scale phase separation that can be easily accessed by conventional lithographic fabrication processes. It is found that by reducing a single-crystal LPCMO thin film to a wire with a width comparable to a scale on the order of the inherent EPS, the system exhibits ultrasharp jumps in resistivity, as shown in Figure 4[27]. These jumps are attributed to a reduction of the transport lanes to a single channel. As the insulating barriers of the charge-ordered state are broken by the reduction of temperature or an increase in magnetic field, the resistance in the wire shows sharp jumps around the MIT, which reflects the nature of the first-order phase transition between ferromagnetic metal and charge-ordered insulator domains. Since the transport measurement can reveal the signature of phase transition of an individual EPS domain in a manganite wire, it becomes possible to probe the EPS domain dynamics of manganites. This is accomplished by setting an LPCMO wire at or very near the critical point of phase transition and measuring the phase fluctuation with high-time resolution. The very limited number of EPS domains that can be hosted in the wire effectively removes the problems associated with spatial averaging methods in the conventional transport measurements while allowing for a high temporal resolution. At the critical point of the MIT, single-domain fluctuations will show a clear signature in time-dependent resistivity measurements, as shown in Figure 5[29]. In Figure 5a, the resistivity (*ρ*) of a 10 μm × 50 μm × 70 nm LPCMO wire under a 3.75-T magnetic field exhibits an ultrasharp jump at the MIT centered at 83 K. This is contrasted with the same sample in a film geometry (Figure 5a, inset) which shows a smooth transition from metallic to insulating behavior across a 150-K window. The extremely large jump in the resistivity of the wire results solely from its geometry’s ability to remove the effects of spatial averaging in transport measurements. By setting the temperature of the LPCMO wire precisely in the middle of the 2-K window found in the temperature-dependent resistivity scan, it is possible to study the microscopic details of the transition in both space and time. Figure 5b shows the time-dependent resistivity while the wire is held at the transition temperature. It is clear that the apparent two-state system with resistivity jumps is actually comprised of a much richer multistate system. There are three inherent resistivity levels, each containing a further two-state fluctuation. However, in unconfined structures such as film (inset in Figure 5) or bulk counterpart, only white noise is observed. These data clearly show that the fluctuations that change the electrical resistance exist in these phase-separated manganite wires. It is observed that these fluctuations exist only near the transition temperature where electronic domains are fluctuating between FMM and COI and are not individually observable in films or bulk transport experiments. Therefore, the fluctuations in the wire are the direct signal of the microscopic fluctuations in EPS domains at the transition temperature. The comparable dimensions of the inherent domains to the wire result in a large change in the total wire resistance when a single domain fluctuates from one phase to another. Not only did these findings give us new insights into the mechanisms that drive electronic phase transitions, but they also open the door to engineering novel devices and could be applied as an on-chip digital randomizer as one example. Recently, large aspect-ratio (length-to-width >300) single-crystal nanowires of La_2/3_Ca_1/3_MnO_3_ were also fabricated by combined optical and focused ion beam lithographies, which preserved their functional properties [66]. Remarkably, an enhanced magnetoresistance value of 34 % in an applied magnetic field of 0.1 T in the narrowest 150-nm nanowire was obtained. Such behavior is ascribed to the strain release at the edges together with a destabilization of the insulating regions. This opens new strategies to implement these structures in functional spintronic devices.

**Figure 6:**
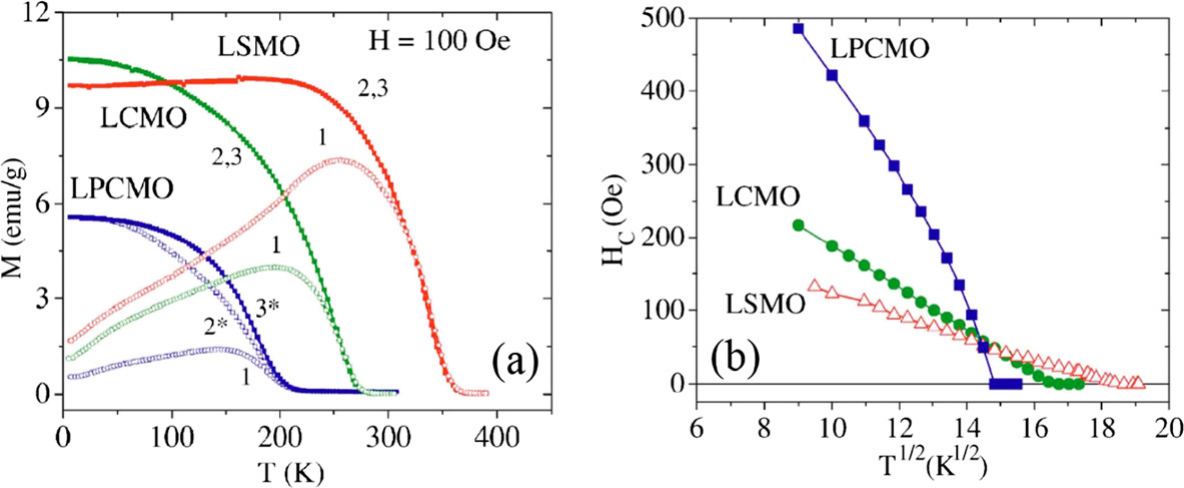
**Magnetizations and square-root temperature dependence of the LSMO, LCMO, and LPCMO nanotubes. (a)***M* vs *T* at 100 Oe of LSMO, LCMO, and LPCMO nanotubes after different magnetothermal processes [54]. The numbers 1, 2, and 3 show the data collected in a 1 ZFC warming process after cooling with zero magnetic field, 2 FCC cooling process with a magnetic applied field of 100 Oe, and 3 FCW warming after the FCC process with 100 Oe. The asterisk indicates that the FCC and FCW in the LPCMO-nanotubes are different. **(b)** Square-root temperature dependence of the coercive fields for the LCMO, LSMO, and LPCMO nanotubes.

In addition to the manganite nanowires, the EPS in the manganite nanotubes are also investigated. Nanotubes are different from nanowires because they typically have a hollow cavity, whereas nanowires are completely filled with nanomaterials. From a viewpoint of device engineering and theoretical considerations, manganite nanotubes with nonplanar geometries have a variety of applications for nanoelectronic components [67-69], transducers, or sensors [70,71]. Recently, perovskite rare-earth manganese tubes such as La_0.67_Sr_0.33_MnO_3_ (LSMO), La_0.67_Ca_0.33_MnO_3_ (LCMO), and La_0.325_Pr_0.300_Ca_0.375_MnO_3_ (LPCMO) have been fabricated using a sol–gel template synthesis process [53,72,73]. Their typical length is about 6 to 8 μm and the average wall thickness is 45, 60, and 150 nm for LSMO, LCMO, and LPCMO, respectively [54]. The walls of the tubes are composed of magnetic nanograins, and their sizes are less than the critical size for multidomain formation in manganites. As a consequence, each particle that constitutes the nanotube walls is a single magnetic domain. Figure 6a shows the magnetizations of the LSMO, LCMO, and LPCMO nanotubes as a function of the temperature *T* measured at different applied magnetic fields (only show the data measured at *H* = 100 Oe) following the next protocol: zero-field cooling (ZFC) (1 in Figure 6a), cooling the sample from the highest *T* with H = 0 Oe; afterward, a magnetic field of H =100 Oe was applied and the magnetization data were collected increasing *T*. Field cool cooling (FCC) (2 in Figure 6a) is performed by measuring the magnetization by cooling the sample with *H* =100 Oe [54]. Finally, in field cool warming (FCW) (3 in the same plot), the system is warmed with *H* =100 Oe after FCC. It was noticed that there exists differences between the FCC (2*) and FCW (3*) curves in a broad temperature range for LPCMO nanotubes. Figure 6b displays the square-root temperature dependence of the coercive fields for the LCMO, LSMO, and LPCMO nanotubes [54]. Clearly, the coercive fields of the LCMO and LSMO nanotubes followed a linear dependence with the square root of temperature, whereas a nonlinear dependence was observed in LPCMO nanotubes, and the higher coercive field value was associated with the competition between the CO and the FM phases in the phase separated LPCMO nanotubes. Normally, a linear dependence is expected in the noninteracting particle systems, which can originate in the single magnetic domains that constitute the walls of the ferromagnetic nanotubes [74]. Therefore, as shown in Figure 6, the LSMO and LCMO nanotubes present a homogeneous ferromagnetic behavior below 340 and 258 K, respectively. The magnetic dead layer avoids the exchange interaction between the nanograins, but the dipolar interaction between them was detected which suggests a fanning array of magnetic moments along the tube axis. The coercive field temperature dependence indicates the presence of weak interactions. As for the LPCMO nanotubes, they became mainly ferromagnetic below 200 K. Their thermal hysteresis and the low magnetization values indicate the presence of an extra charge-ordered phase in the LPCMO nanotubes. The small grain size may generate small charge-disordered regions, which are easily transformed to a ferromagnetic region by field or increasing/decreasing temperature. Also, the charge-disordered phase attenuates the interaction between single magnetic domains when this phase is reduced by the application of a magnetic field; the system increases its ferromagnetic character. So, the control of the charge-disordered phase fraction could be used to tune the magnitude of the interaction between the single magnetic domains which affects the coercive fields.

### EPS in manganite nanostructured films/patterns

**Figure 7:**
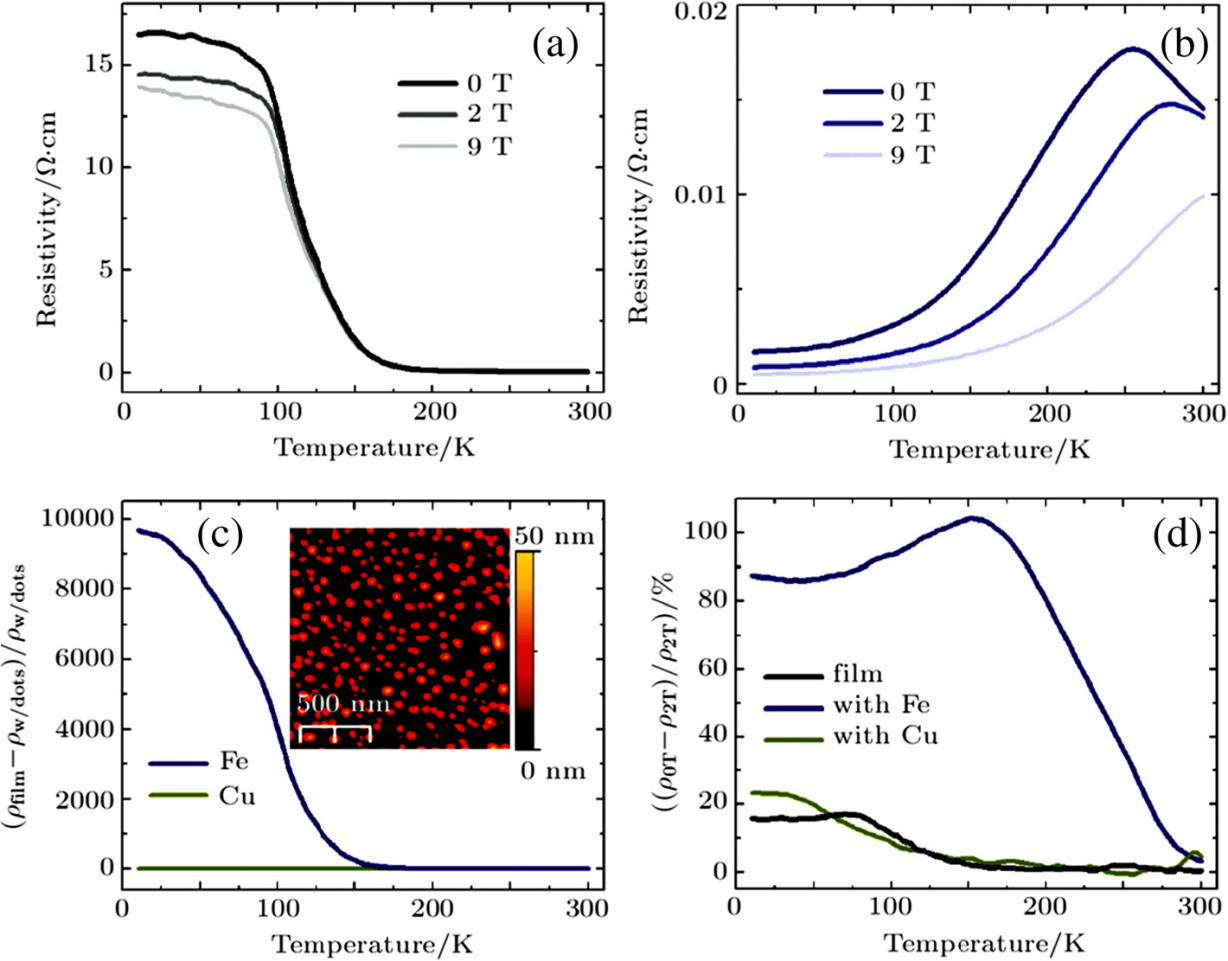
**Transport properties of ultrathin LCMO film before and after application of nanodots [**[75]**]. (a)** Resistivity behavior for 20-nm ultrathin film of La_0.7_Ca_0.3_MnO_3_ showing insulating behavior and no clear metal-insulator transition. **(b)** Resistivity data of the same film after applying Fe nanodots to surface showing a recovery to bulk-like behavior with an MIT temperature of 255 K at 0 T (note change in scale). **(c)** Ferromagnetic Fe nanodots drive a huge change in the film’s resistivity compared to the diamagnetic Cu nanodots. Insets: AFM images of typical nanodot coverages for Cu and Fe systems on LCMO films. **(d)** Magnetoresistive behavior shows a much higher magnetic response in the spin coupled system.

**Figure 8:**
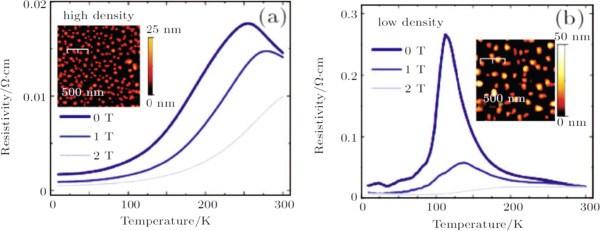
**Comparison of transport properties with different Fe nanodot density.** Resistive data for an ultrathin LCMO film after application of low density Fe nanodots shows recovery of the metal-insulator transition but with a much lower transition temperature than that seen at higher nanodot densities [75].

In most CMR manganites, both the MIT and the amplitude of magnetoresistance are critically dependent upon the percolation of ferromagnetic metal domains in the system. Controlling the formation and the spatial distribution (size, density, symmetry, etc.) of the electronic domains will not only help to understand the origin of the EPS but also help to design manganites or other correlated electronic materials with desired properties for all-oxide-based electronic devices. Recently, a novel method called electronic nanofabrication (a conceptually new approach) is developed to control the formation and the spatial distribution of electronic domains in manganites [35]. In contrast to the conventional nanofabrication, the electronic nanofabrication patterns electronic states in materials without changing the actual size, shape, and chemical composition of the materials, which is a promising method for manganites. For example, magnetic Fe nanodots are grown on the surface of a 20-nm-thick La_0.7_Ca_0.3_MnO_3_/LaAlO_3_(001) film, which could turn the film from an insulator to a metal with a high MIT temperature, as shown in Figure 7[75]. The underlying mechanism is understood to be the local magnetic exchange field between Fe and Mn spins that aligns the local Mn spins leading to the formation of a local metallic state. As shown in Figure 8, the MIT temperature can be also tuned by the density of Fe nanodots, which strongly indicates that the local metallic state follows the spatial locations of the Fe nanodots [75]. Besides the electronic nanofabrication technique, other methods such as atomic force microscopy lithography [28], electron-beam lithography (EBL) [76-80], focused ion beam (FIB) milling [33,34,81-84], and chemical growth and etching [85,86] are also used to fabricate manganite nanostructured patterns from oxide thin films. For example, microscopic Pr_0.65_(Ca_0.75_Sr_0.25_)_0.35_MnO_3_ (PCSMO) thin films were fabricated into patterns by EBL with width comparable to the length scale of EPS (~1 μm), where spontaneous resistance jumps along with the local Joule heating-induced step-like negative differential resistance were clearly observed [76]. Recently, LCMO microbridges with different widths were also fabricated by EBL method, where the MIT temperature was found to be decreased as reducing the bridge width, and the MIT even disappeared over the measured temperature range for the bridge with a width of 500 nm [76]. The underlying mechanism for this phenomenon is the confined geometry, which is dominated by the filamentary conduction mechanism. The magnetoresistance of the bridge also shows interesting behavior for enhanced *e*-*e* interactions in the presence of spin disorder; it can decrease and even change its sign in the bridges with widths of 1.5 and 1.0 μm under magnetic field of 1 T*.* The obvious size effects in the manganite microbridge nanopatterns are invaluable for further understanding the EPS phenomenon and its role in CMR effect.

### Origin of EPS in perovskite manganite nanostructures

**Figure 9:**
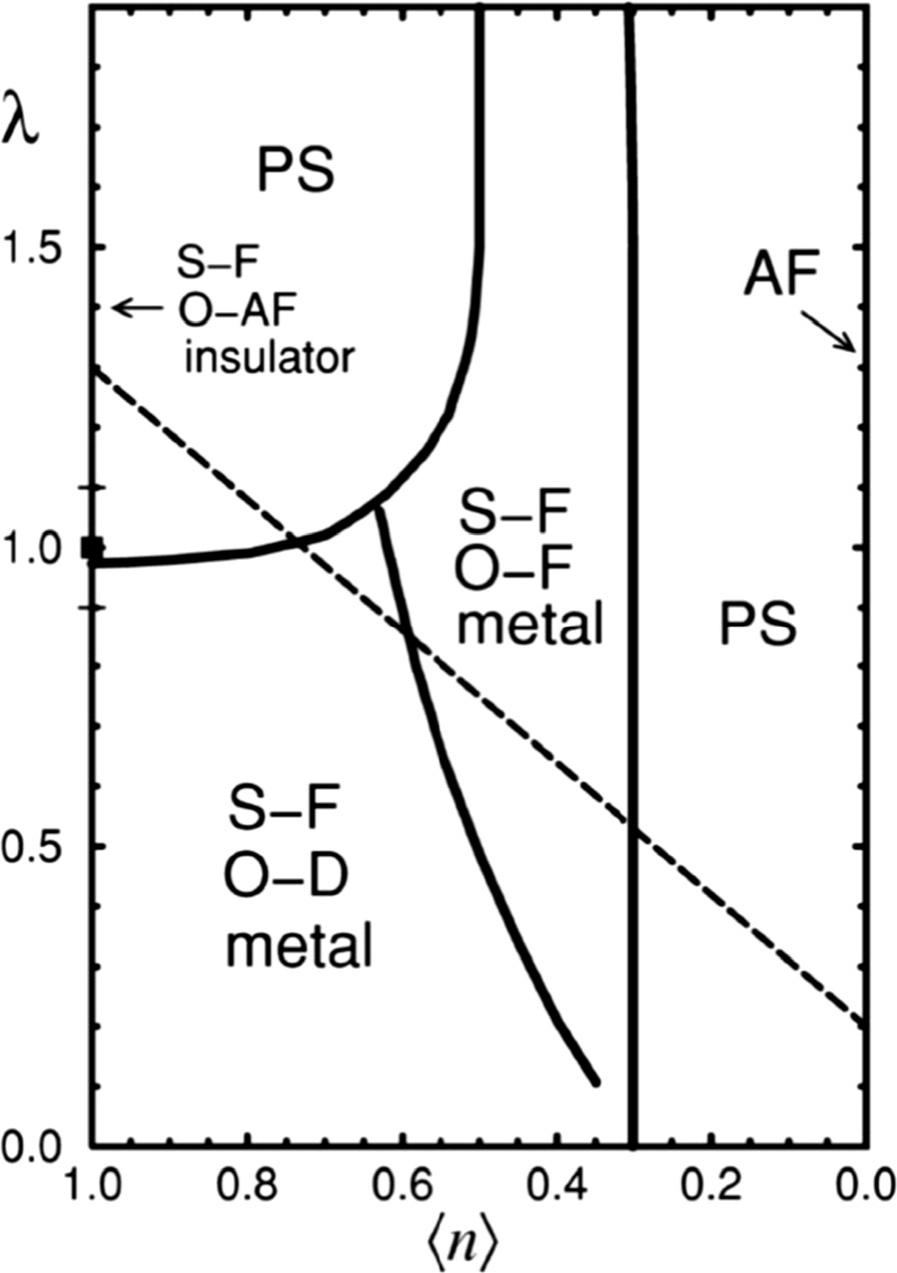
**Phase diagram of two-orbital model in one-dimensional and *T *~0 including Jahn-Teller phonons, obtained with Monte Carlo techniques [**[90]**].** S-F labels a spin-ferromagnetic configuration. O-F, O-AF, and O-D denote a state where the orbital degrees of freedom are ordered uniformly, staggered or they are disordered, respectively; PS indicates a phase separated state, and AF in an antiferromagnetic state. The Hund-coupling is *J*_H_ = 8, the Heisenberg coupling between localized classical spins *J*_AF_ = 0.05, both in units of the hopping amount the same orbitals.

EPS as an inherent electronic inhomogeneity has been observed in real space with atomic-scale resolution in the perovskite manganites, which is generally regarded to be crucial for the CMR effect. This greatly stimulates a growing and theoretical interest in the EPS of perovskite manganite nanostructures. Now, the main theoretical approaches developed for investigating the EPS in perovskite manganite nanostructures can be classified into two categories, namely, approaches based on the model Hamiltonians and phenomenological theory. Dagotto and colleagues have developed one-orbital FM Kondo model and two-orbital model with Jahn-Teller phonons to investigate the EPS phenomenon in one-dimensional manganites [58,87-89]. In the model of Kondo lattice Hamiltonian with ferromagnetic Hund's coupling, the classical limit for the spin of the (localized) t_2g_ electrons is analyzed on lattices of dimension 1, 2, 3, and ∞ using several numerical methods [60,87,88]. They found that the expected double-exchange-induced strong tendencies to ferromagnetic correlations at low temperatures were in competition with a regime of phase separation, which occurred between the hole-undoped antiferromagnetic and hole-rich ferromagnetic regions. Although, the one-orbital model for manganites contains interesting physics, notably, a FM-AF competition that has similarities with those found in experiments. However, to explain the notorious orbital order tendency in Mn-oxides, it is crucial to use a model with two orbitals, where there is an electron Jahn-Teller phonon coupling and also Coulomb interactions [89,90]. Under the assumption that both localized e_g_-spins and phonons are classical, the model without Coulombic terms can be studied fairly accurately using numerical and mean-field approximations. The calculated results for a one-dimensional system at low temperature by considering the two e_g_ orbitals and the Jahn-Teller phonons enrich the phase diagram considerably, as shown in Figure 9[90]. Obviously, the phase diagram is much rich, which includes different phases such as metallic and insulating regimes with orbital order. It is clear that phase separation appears at small e_g_-densities between an electron-undoped AF-state and a metallic uniform-orbital-ordered FM state. Ahn et al. also proposed a model Hamiltonian including electron–phonon interactions and long-range elastic coupling between the local lattice distortions [91]. They presented a scenario for mesoscopic/microscopic inhomogeneities and suggested them to be the main source of the CMR effect. Since the physics of perovskite manganites is controlled by many degrees of freedom at the atomic level and the associated energy scales, Ramakrishnan et al. also developed a microscopic model for manganites that includes all the important energy scales present in them [92]. In this model, the degeneracy of the two e_g_ orbitals is split into two types of states, *l* and *b* at each manganese site by electron-lattice coupling. The *l* state is polaronic and has an exponentially reduced hopping amplitude, whereas the electrons within the *b* states hop with the bare amplitude. Such two-fluid model of manganites demonstrates colossal magnetoresistive response and reproduces the physical transport properties confirmed by the experimental measurements. Due to the local strong Mott-Hubbard repulsion, the simultaneous occupation at both the *l* and *b* states on a given site is not allowed; this model exhibits macroscopic phase separation, where the region with *l* polarons corresponds to the charge-ordered states, while that with *b* electrons corresponds to the FM metal. However, due to the frustration of the long-range Coulomb interactions, the phase separation usually exhibits as a structure of nanoscopic FM metallic domains with interspersed domains of polaron Coulomb glass, similar to the charge-ordered FM state revealed by the electron holography [11]. Therefore, the manganites are intrinsically inhomogeneous at length scales of nanometers due to the strong electronic correlations. A phenomenological Ginzburg-Landau theory approach is also developed by using a Landau free-energy function and introducing the term of electronic softness to rationalize the possibility of phase coexistence and electronic inhomogeneities [93]. In this approach, magnetic and charge modulations are argued to coexist in new thermodynamic phases in contrast to the previous models where the phase separation originates from disorder or as a strain-induced kinetic phenomenon. This approach leads to a rich diagram of equilibrium phases, qualitatively similar to those seen experimentally. The success of this approach argues for a fundamental reinterpretation of the nature of charge modulation in manganite materials, from a localized to a more extended ‘charge-density wave’ picture. The same symmetry considerations that favor textured coexistence of charge and magnetic order may apply to many electronic systems with competing phases. The resulting ‘electronically soft’ phases of matter with incommensurate, inhomogeneous, and mixed order may be general phenomena in correlated systems.

Since a number of competing energy scales are operative in manganite oxides giving rise to a large number of electronic orders such as spin, charge, and orbital (and associated lattice order), the emergence of these orders and the associated couplings between them should be considered in a full Hamiltonian model for manganites, which makes the theoretical understanding of the EPS quite complex. Much work is further needed in this challenging area of research.

## Conclusions

In recent years, a remarkable progress has been achieved in understanding the EPS phenomenon in low-dimensional perovskite manganite nanostructures such as manganite nanoparticles, nanowires, nanotubes, and nanostructured films/patterns. This progress is mainly made possible by building upon the experimental measurements and theoretical approaches, and clearly establishes the phase completion as the main source of the CMR effect in manganite oxides. The shape and scale of EPS are different for different systems with electronic domain sizes ranging from a few nanometers to several micrometers. However, the microscopic nature of the EPS in perovskite manganite oxides and the physical origin of the large length-scales that arise in these systems are still unclear although much theoretical work based on model Hamiltonians and phenomenological models has been done. Clearly, much further work is needed to be done on both the experimental and theoretical fronts to understand the nature of the EPS manganite oxides, especially at the nanoscale. On the experimental side, a new technique is needed to be developed to control the formation and the spatial distribution of electronic domains in manganite oxides, which should allow to simultaneously probe EPS domains with different electronic states and give the vital information on phase formation, movement, and fluctuation. Such a novel technique called electronic nanofabrication has been developed. In striking contrast to the conventional nanofabrication, the electronic nanofabrication patterns electronic states in materials without changing the actual size, shape, and chemical composition of the materials, which allows one to control the global physical properties of the system at a very fundamental level and greatly enhances the potential for realizing true oxide electronics. The theorists need to quantitatively clarify the electronic properties of the various manganite phases based on microscopic Hamiltonians, including strong electron–phonon Jahn-Teller and/or Coulomb interactions. Thus, quantitative calculations for addressing the CMR effect help us to better understand the physical nature of EPS phenomenon. However, to get a full understanding of the EPS phenomenon in low-dimensional manganite nanostructures, much work remains to be done for realizing its practical applications in oxide electronics.

## Competing interests

The authors declare that they have no competing interests.

## Authors’ contributions

LL and XZ designed the structure and modified the manuscript articles; LL drafted the manuscript. HW participated in the sequence alignment. All authors read and approved the final manuscript.
